# The role of bullying victimization in youth suicide: A scoping review of empirical evidence

**DOI:** 10.1186/s12887-026-07299-z

**Published:** 2026-07-15

**Authors:** Anne. C. Frederiks-Franssen, Rozemarijn van der Ploeg, Antje Janshen, Saskia Mérelle, Diana D. van Bergen

**Affiliations:** 1 Department of Pedagogy and Educational Sciences, Faculty of Behavioural and Social Sciences, University of Groningen, Groningen, The Netherlands; 2 Department of Social Work, Rotterdam University of Applied Sciences, Rotterdam, The Netherlands; 3 Department of Clinical Psychology and Experimental Psychopathology, Faculty of Behavioural and Social Sciences, University in Groningen, Groningen, The Netherlands; 4 Research Department, 113 Suicide Prevention, Amsterdam, The Netherlands; 5 Department of Child and Adolescent Psychiatry and Psychosocial Care, Amsterdam UMC, Amsterdam, The Netherlands

**Keywords:** Bullying victimization, Youth suicide, Cyberbullying, Suicide risk factors

## Abstract

**Background:**

While bullying victimization is a well-established risk factor for suicidal ideation and nonfatal suicide attempts among young people, its role in suicide deaths remains poorly understood. This scoping review synthesizes empirical evidence on the prevalence and role of bullying victimization among children and adolescents who died by suicide.

**Methods:**

Following PRISMA guidelines, peer-reviewed empirical studies were included if they examined bullying victimization during childhood or adolescence (≤ 21 years) among individuals who died by suicide. Searches were conducted across seven databases in October 2023 and October 2025. Of the 4,233 screened records, 32 studies met the inclusion criteria after full-text review and quality appraisal using the QUIPS and CASP tools. The included studies employed record-based (60%), qualitative narrative (28%), longitudinal cohort (6%), and online ethnographic (6%) designs.

**Results:**

Among studies that systematically assessed bullying victimization, 29–55% reported its presence in the lives of deceased youth, compared with 0.1–22% in studies where bullying was documented only when spontaneously mentioned by informants. Record-based studies indicated that bullying victimization was more frequently documented among females, younger adolescents, and LGBTQ+ youth. Longitudinal cohort studies demonstrated that childhood bullying victimization increased the risk of suicide in adulthood, even after adjusting for individual and familial characteristics, although effect sizes were modest.

Qualitative studies, often employing psychosocial autopsy methods, described bullying victimization as a persistent psychosocial stressor embedded within broader constellations of adversity, contributing to feelings of sadness, entrapment, and lack of belongingness. Online ethnographic studies found that hostile audience responses during live-streamed suicides function as cyberbullying and may exacerbate humiliation and hopelessness during acute crises.

**Conclusions:**

Bullying victimization is an important yet understudied risk factor in youth suicide. Across study designs, it emerged as a long-term vulnerability of suicide embedded within complex psychosocial interactions. However, methodological limitations and inconsistent assessment constrain firm conclusions and calls for systematic, theory-driven, and technologically informed research to clarify the contribution of bullying victimization to suicide risk across development.

**Supplementary Information:**

The online version contains supplementary material available at https://doi.org/10.1186/s12887-026-07299-z.

## Background

Worldwide, suicide is the third leading cause of death among adolescents [1]. Among young people aged 10 to 19 years, an estimated global average of 3.7 per 100,000 die by suicide [2]. However, suicide rates vary considerably across countries and regions, with markedly higher rates reported in countries such as the United States, Australia, and several Eastern European countries [3]. Accordingly, the World Health Organization (WHO) has identified suicide as a serious global public health issue, and reducing suicide mortality has been listed as a priority for prevention by the WHO. Suicide is a multifaceted public health challenge arising from a complex interplay of factors, which vary on an individual basis [4]. Among these, bullying victimization has been found to be a prominent risk factor for suicide-related outcomes among adolescents, including suicidal ideation, suicide attempts, and non-suicidal self-injury [5–10].

Regarding suicidal ideation, a meta-analysis of longitudinal studies identified a robust predictive relationship between bullying and subsequent suicidal ideation, indicating a significant association between bullying victimization and the development of suicidal thoughts over time [11]. In addition, a comprehensive systematic review and meta-analysis of both cross-sectional and longitudinal studies revealed a robust association between bullying victimization and suicidal outcomes [12] Children and adolescents exposed to bullying were found to have a significantly increased risk of suicidal ideation and suicide attempts, with evidence of dose–response effects indicating higher risk with more frequent victimization. These findings primarily concern nonfatal suicidality outcomes, leaving open the critical question of how bullying victimization plays a role in youth suicide deaths. At the same time, they provide important insights with regard to the prevention of suicide death: a suicide attempt is one of the strongest predictors of suicide [4, 13–15], and suicide attempts are often preceded by suicidal ideation and/or suicidal self-injury [14]. However, despite these important insights, the existing evidence remains primarily based on nonfatal outcomes rather than suicide deaths.

Importantly, within theoretical frameworks such as the Ideation-to-Action Framework, suicidal ideation is conceptualized as a distinct cognitive phase that does not necessarily progress to suicidal behavior, which involves the transition to action [16, 17]. This distinction suggests that risk factors for the emergence of suicidal thoughts may differ from those involved in the progression to suicide attempts or suicide deaths. This perspective emphasizing unique phases in the suicidal process is also reflected in several leading theoretical frameworks of suicide, including the Interpersonal Theory of Suicide (IPS), the Integrated Motivational–Volitional Model (IMVM), and the Cry of Pain Model [18–20]. These frameworks offer a useful lens for understanding how bullying victimization may contribute to pathways leading to suicide among adolescents. Within these models, bullying is conceptualized as a social stressor that may increase vulnerability to suicidal ideation through mechanisms such as social exclusion, perceived burdensomeness [18], and feelings of defeat or entrapment [19, 20]. These mechanisms have been identified as key drivers of suicide-related outcomes, consistent with broader evidence demonstrating the pervasive psychological impact of bullying victimization on adolescent mental health [18–21].

To further contextualize the role of bullying victimization as a risk factor, it is important to consider both its conceptualization and its potential psychosocial consequences. Bullying is characterized by three core features: repetition, intentionality, and an imbalance of power [22, 23]. Previous studies indicate that various aspects of bullying victimization are associated with suicidal behavior, including being the victim of social exclusion, verbal and physical abuse, and peer coercion or manipulation [11, 24–27]. It has been suggested that being bullied may lead to a range of interconnected challenges, such as psychological trauma, substance abuse, and low self-esteem, thereby increasing adolescents’ susceptibility to suicidal behavior [11, 12, 24–26].

These emotional detrimental processes and the negative mental health consequences of bullying victimization may be further intensified in the digital age, where traditional forms of bullying have evolved alongside new technological developments. The rise of social media and digital communication platforms has facilitated the emergence of cyberbullying, a form of bullying that transcends physical spaces and extends into an adolescent’s online world. Although cyberbullying shares core characteristics with traditional bullying – namely repetition, intentionality, and an imbalance of power – it differs in at least four ways that may amplify its impact [24, 27, 28]. First, cyberbullying can reach a wider audience than traditional forms of bullying, thereby intensifying perceived humiliation [24]. Second, perpetrators may remain anonymous in online contexts [28, 29]. Third, cyberbullying can occur anywhere and at any time [24, 30]. Finally, even a single incident can be repeatedly shared online, rendering the bullying potentially permanent in its visibility [31].

Although research has established associations between both traditional and cyberbullying victimization and suicidal thoughts and behaviors [27], findings regarding their relative impact on youth suicide remain inconclusive [9, 32]. Crucially, this body of work has focused almost exclusively on nonfatal outcomes, and the role of traditional and/or cyberbullying victimization in youth suicides remains insufficiently understood.

Against this background, an important discrepancy emerges between scientific evidence and public narratives surrounding youth suicide. This gap in academic research contrasts sharply with media portrayals, which in recent decades have frequently framed youth suicides as the direct and/or sole consequence of bullying victimization, as illustrated by the reporting on Canadian teenager Amanda Todd in 2012 [33]. Headlines such as *“Canadian teen found dead weeks after posting wrenching YouTube video detailing bullying”* [34] framed the suicide of this 15-year-old girl as a direct consequence of being bullied. More recent headlines reflect a similar narrative. For instance, media reports often describe youth suicides as occurring “after being bullied” or following “relentless” victimization [35–38]. Although media coverage often emphasizes bullying victimization as an immediate risk or precipitating factor for suicide, scientific research has yet to establish a comprehensive understanding of its role in cases of youth suicides.

Taken together, these findings highlight a critical gap in the literature regarding the role of bullying victimization in youth suicides, despite theoretical models suggesting a substantial impact. To address this gap, the present scoping review systematically maps and provides a structured synthesis of empirical evidence on the role of bullying victimization in cases of suicide among children and adolescents. Specifically, it addresses two research questions: (1) to what extent bullying victimization is identified among youth suicide cases within the included studies, without aiming to estimate population-level prevalence, and (2) what role bullying victimization in these developmental periods may play as a risk factor for youth suicides.

Given the exploratory nature of the research questions and the heterogeneity of the available literature, a scoping review methodology was considered most appropriate as youth suicide is a relatively rare outcome, and the available evidence is limited. Scoping reviews are well suited to systematically map the breadth and characteristics of fragmented bodies of literature, especially in areas that are not yet amenable to quantitative synthesis [39, 40]. The current study uses this approach to allow for a broader examination of how bullying victimization is identified and conceptualized in relation to suicide deaths.

## Methods

This scoping review was conducted following PRISMA guidelines, with an emphasis on transparency and completeness (see Supplementary File 1: PRISMA Checklist). A structured approach was employed to enhance the study’s transparency and replicability [41].

### Inclusion and exclusion criteria

Each selected study was required to report empirical data, and bullying victimization had to be explicitly addressed in the empirical research section. Suicidal behavior as an outcome was required to be fatal. Preference was given to peer-reviewed studies and committee-approved dissertations; however, reports offering substantive insights into the topic were also considered, regardless of peer-review status. To ensure a minimum level of methodological rigor, all included studies were assessed for methodological quality using the CASP checklist for qualitative studies and the QUIPS tool for quantitative studies. Studies with critically low methodological quality were excluded based on these assessments.

Given our focus on adolescence, studies including individuals who died by suicide in adulthood were considered only if the bullying occurred during childhood or adolescence, up to and including age 21. This cut-off reflects the view of adolescence and emerging adulthood as a developmental continuum in which psychosocial and identity-related vulnerabilities remain salient [42]. It also aligns with definitions used by international health organizations, which typically define adolescence as ages 10–19, while broader categories such as “youth” or “young people” often include individuals up to age 24 [43]. Setting the boundary at age 21 allowed us to capture studies addressing late adolescence while ensuring that experiences of bullying victimization fell within the broader developmental period of childhood and adolescence, prior to full adulthood. While some definitions extend youth up to age 24, a more conservative cut-off at age 21 was chosen to maintain a clearer focus on adolescence and to reduce heterogeneity associated with developmental differences in young adulthood.

Given the study’s central focus on bullying victimization among adolescents – defined as peer-to-peer interactions – (sexual) abuse was included only if it occurred between adolescents; in such cases it was categorized as a form of bullying [44]. Similarly, studies involving games or online challenges [45–47] were included only if the bullying characteristics of manipulation or peer pressure were evident.

We did not restrict our search to English-language publications, as substantial suicide research has been conducted in diverse global contexts [7, 25, 48–52]. Abstracts and, when necessary, full-text articles were translated with the assistance of an AI-based translation tool (DeepL).

### Search strategy

Following preregistration [53], the initial search was conducted on October 26 and 27, 2023, and updated on October 15, 2025. The following databases were searched: Web of Science, PsycINFO, MEDLINE, Psychology and Behavioral Sciences, CINAHL, ERIC, and SocINDEX. Boolean operators were used to combine search terms related to (1) “suicide death” and (2) “bullying victimization,” as presented in Table 1. In defining search terms related to bullying victimization, expert consultation was undertaken to ensure comprehensive coverage, particularly with respect to online forms of bullying. To ensure that suicide was the primary focus of retrieved studies, suicide-related terms were required to appear in the title, abstract, or subject headings. By contrast, terms related to bullying victimization were searched in the title, abstract, subject headings, or full text, reflecting the conceptualization of bullying victimization as one of multiple potential contributing factors within the complex interplay of determinants leading to suicide. After removing duplicates, the search yielded a total of 4,233 articles.

**Table 1:** Search terms

Search terms	Search string
Suicide death	“Suicide*” OR “selfkill*” OR (self-destructive NEAR/2 behavio*) OR “self-poison*” OR (self NEAR/2 stabb*) OR (self-directed NEAR/2 violence)
Bullying victimization	“Bully*” OR “bulli*” OR “mobb*” OR “harassment” OR “sexting” OR “sextortion” OR (cyber NEAR/2 abuse) OR (cyber NEAR/2 aggression) OR (online NEAR/2 aggression) OR (internet NEAR/2 aggression) OR (electronic NEAR/2 aggression) OR (peer NEAR/2 victimization*) OR (cyber NEAR/2 victimization*) OR “cyberstalking” OR “cyberbully*” OR “cyberbulli*” OR “happy slapping” OR “doxing” OR “blue whale challenge” OR (internet NEAR/2 abuse) OR (online NEAR/2 abuse) OR (online NEAR/2 sexual abuse) OR (internet NEAR/2 sexual abuse)

### Eligibility assessment

Two researchers independently screened the titles and abstracts of the identified 4,233 articles to assess their eligibility according to the predefined inclusion and exclusion criteria. To evaluate the consistency and rigor of the screening process, 25% of all records were independently screened by a third researcher. Discrepancies were resolved through discussion among the two or three researchers involved.

The subsequent step involved retrieving full texts for articles that received conflicting ratings or could not be adequately assessed based on the title and abstract (*N* = 383). Disagreements regarding inclusion were resolved through discussion until consensus was reached. During this phase, one Japanese-language article could not be translated by AI and was therefore excluded. After full text screening, 49 articles were retained.

The methodological quality of these 49 studies was then assessed in accordance with the predefined quality assessment criteria. Eight studies were excluded due to critically low methodological quality. An additional 11 studies were excluded after closer inspection for failing to meet the inclusion criteria, resulting in a final sample of 30 eligible studies.

### Additional search strategy

An additional search strategy was employed. Reference lists of the eligible studies were examined, yielding six potentially relevant articles. Following discussion among three researchers, three of these were excluded due to insufficient conceptualization of bullying, and one was excluded because of poor methodological quality. This process resulted in two additional studies being included.

## Results

**Fig. 1:**
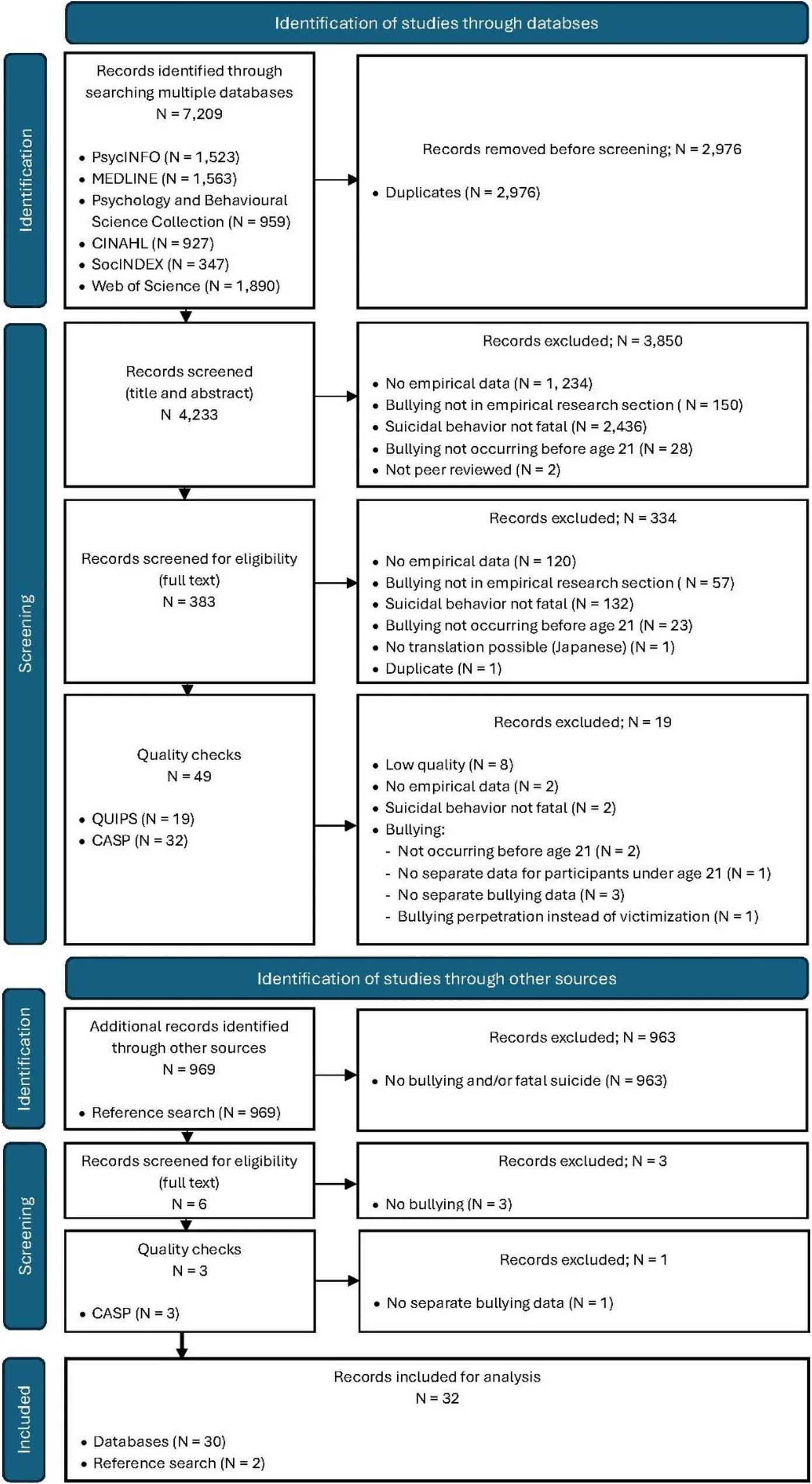
Flow-chart of included articles

In total, 32 publications were included in the review (Fig. 1). Most were published in English; one Russian-language article was translated using AI-assisted translation (DeepL). The included studies originated from a wide range of countries: Canada, the United States, the United Kingdom, Norway, Sweden, the Netherlands, the Russian Federation, Japan, Korea, China, and Australia (see Table 2). No grey literature reports were ultimately included, as none met the inclusion criteria.

**Table 2:** Characteristics and main findings of studies examining bullying victimization in relation to youth suicide

Study		Method				Report of bullying			Sample					Main findings		
Reference	Country	Datasets	Design	Type of data and measures used	Informants	Form	Systematically		Cases	Age	Number (*N*)	Period		Frequency	Role	
**RECORD BASED STUDIES**																
Affleck et al. (2021) [69]	Canada		Comparative record based study	Coroner/medical examiner and healthcare records	Coroners/medical examiners	Recorded only if documented in records	No		Suicide deaths compared to suicide attempts, suicidal ideation	Not speci-fied	63	2014–2018		5–24% (age-dependent)		
Cina et al. (2025) [68]	United States of America		Record based study	Medical examiner, police, toxicology records	Coroners/medical examiners; police	Recorded only if documented in records	No		Firearm-related suicide deaths	0–18	36	2016–2021		6%		
Clark et al. (2020) [80]	United States of America		Record based study	National Violent Death Reporting System (NVDRS) multi-source records	Coroners/medical examiners; police; family members	Recorded only if documented in records or communications	No		Suicide deaths	10–19	9,884	2003–2017		5–21% (LGBTQ status)	Bullying victimization was reported more frequently among LGBTQ youth, particularly at younger ages.	
DiVietro et al. (2024) [70]	United States of America		Record based study	NVDRS multi-source records	Coroners/medical examiner; police	Recorded only if documented in records	No		Firearm-related suicide deaths	10–17	1,112	2014–2018			School bullying victimization was reported as a recurring theme in suicides among younger children (10–14 years).	
Duffy et al. (2024) [71]	United States of America		Comparative record based study	National Fatality Review–Case Reporting System (NFR-CRS); multi-source records	Medical examiners/coroners; police; first responders; healthcare professionals	Recorded only if documented in records	No		Suicide deaths, SDG-youth compared to non SDG-youth	0–19	323	2015–2020		15–28% (sexual and gender diversity)	Bullying victimization was more frequently reported among sexual- and gender-diverse youth and was associated with suicide in this group.	
Grøholt & Ekeberg (2003) [75]	Norway		Comparative record based study	Medico-legal administrative records	Coroners/medical examiners; police; family, friends, and professionals	Not directly measured; incidental finding.	No		Suicide deaths	≤ 14 vs. 15–19	14/113	1990–1992			In isolated cases, bullying was described as a proximal trigger for impulsive suicide.	
Hill et al. (2021) [67]	Australia		Record based study	National Coronial Information System (NCIS); multi-source records	Coroners/medical examiners; police	Recorded in police or coroner records; coroner data prioritized.	No		Suicide deaths	10–24	3,027	2006–2015		2–5% (sex-dependent)		
Holland et al. (2017) [76]	United States of America		Mixed-methods secondary analysis	NVDRS multi-source records	Coroners/medical examiners; police records; informants	Recorded only if documented in interviews or records.	No		Suicide deaths	11–15	482	2003–2014		9%	Bullying victimization at school typically co-occurred with relational and academic stressors rather than acting as a primary contributor.	
Karch et al. (2013) [65]	United States of America		Record-based study	NVDRS multi-source records	Coroners/medical examiners; police	Recorded only if documented in records	No		Suicide deaths	10–17	1,047	2005–2008		3%		
Lee et al. (2019) [77]	Australia		Record-based study	Coronial records	Coroners/medical examiners; police; healthcare professionals; informants; witnesses	Recorded as part of broader psychosocial stressors.	Un-clear		Suicide deaths	10–19	273	2006–2015		25% (age/sex dependent)	Younger adolescents and females who died by suicide were more likely to have experienced multiple stressors, including bullying victimization.	
Makushkin et al. (2024) [74]	Russian Federation		Record-based study	Health service administrative records	Health care professionals	Recorded only if documented in health service records; data may be incomplete.	No		Suicide deaths and suicide attempts	10–17	429	2023		2% overall, 2% cyber-bullying		
Orlins et al. (2021) [78]	United States of America		Record-based study	NVDRS multi-source records	Coroners/medical examiners; police; witnesses	Recorded only if documented in records	No		Suicide deaths	≤ 17	197	2017		7%	Bullying victimization was rarely identified as the direct precipitant of interpersonal conflict preceding suicide.	
Ream (2020) [66]	United States of America		Comparative record-based study	NVDRS multi-source records	Coroners/medical examiners; police	Recorded only if documented in records	No		Suicide deaths, LGBTQ+ compared to non-LGBTQ+	12–29	788	2013–2016		7–11% (sexual orientation and age)	Bullying victimization was reported more frequently among LGBTQ youth and younger individuals, and less frequently among non-LGBTQ males.	
Rodway et al. (2016) [60]	England	Data-set 2	Record-based national surveillance study	UK multi-agency administrative records (Office for National Statistics (ONS)-linked)	Coroners/medical examiners; police; healthcare professionals	Recorded only if documented in records	No		Suicide deaths	10–19	130	Jan 2014-Apr 2015		6–22% (age-dependent); 6% cyber-bullying; 7% recent bullying	Bullying victimization predominantly involved face-to-face interactions and was more frequently reported among females and younger adolescents; cyberbullying rarely occurred in isolation.	
Rodway et al. (2020) [58]	United Kingdom	Data-set 2	Record-based national surveillance study	UK multi-agency administrative records (ONS-linked)	Coroners/medical examiners; police; healthcare professionals	Recorded only if documented in records	No		Suicide deaths	10–19	544	2014–2016		16–29% (sex-dependent); 5% cyber-bullying	Bullying victimization was reported alongside other common antecedents, including mental illness, self-harm, academic stress, and bereavement.	
Rodway et al. (2023) [59]	United Kingdom	Data-set 2	Record-based national surveillance study	UK multi-agency administrative records (ONS-linked)	Coroners/medical examiners; police; healthcare professionals	Recorded only if explicitly reported by informants or professionals.	No		Suicide deaths	10–19	544	2014–2016		19% overall; 2% exclusively cyber-bullying; 3% recent bullying	Cyberbullying victimization was reported primarily in combination with face-to-face bullying.	
Shiratori et al. (2014) [73]	Japan		Network analysis	National police statistical dataset	Police	Recorded only if documented in police investigation records.	No		Suicide deaths	12–98	2,293	2007–2009		Rare (≤ 1 case)	Bullying victimization showed limited overlap with other suicide motives in network analyses.	
Sinyor et al. (2014) [73]	Canada		Record-based study	Office of the Chief Coroner records	Coroners/medical examiners; police; medical professionals; informants; personal documents	Dependent on informant disclosure; bullying may be underreported.	No		Suicide deaths	10–19	94	1998–2011		8%	Bullying victimization was the sole identified contributing factor in fewer than 5% of cases.	
Valle et al. (2008) [72]	England		Record-based study	Coroner’s inquest records	Coroners/medical examiners	Recorded only if explicitly documented in official records; Subject to definitional ambiguity and reporting variability.	No		Suicide deaths	8–18	77	1996–2002		13%	Young people showed heterogeneous responses to bullying; in a small number of extreme cases (*N* = 2), bullying was identified as the primary contributing factor.	
**STUDIES BASED ON QUALITATIVE INTERVIEWS**																
Balt et al. (2023) [56]	The Netherlands	Data-set 1	Multi-methods psychological autopsy study	Qualitative informant interviews	Parents and peers	School and cyberbullying addressed in informant interviews.	Yes		Suicide deaths	10–19	35	2017		17% overall; 11% cyber-bullying	Threatening or harmful digital messages, including encouragement of self-harm or suicide, were reported in a small number of cases; online sexual victimization was reported in two cases.	
Freuchen et al. (2012) [62]	Norway	Data-set 3	Psychological autopsy study	Informant interviews; questionnaires	Parents	Parent report of bullying; post-interview SDQ.	Yes		Suicide deaths, compared to accidents and community controls	≤ 15	41	1993–2004		29%	No statistically significant difference in reported bullying victimization was observed between suicide and accident groups after adjustment for subthreshold depression.	
Freuchen & Grøholt (2013) [61]	Norway	Data-set 3	Psychological autopsy study	Informant interviews; questionnaires (SDQ); police records; suicide note analysis	Parents; police	Parent report of bullying; post-interview SDQ.	Yes		Suicide deaths, note writers compared to non-note writers	≤ 15	42	1993–2004		21–39% (note-writing status)		
Kour-giantakis et al. (2025) [83]	Canada		Exploratory qualitative study	Online sociodemographic questionnaires; semi-structured informant interviews	Parents	Bullying identified as a qualitative theme in open-ended interviews.	Un-clear		Suicide deaths	≤ 29	17	Jan 2023-Jun 2023			Bullying victimization was reported in school contexts; reports also included experiences of racism and discrimination related to race/immigration status, sexual orientation, and gender identity.	
Lee et al. (2020) [82]	Korea		Record-based study of student suicide cases	Teacher reports and school records; qualitative text analysis	Teachers	Bullying coded only if explicitly mentioned by teachers.	No		Suicide deaths	6–18	417	2011–2017		3% (sex-dependent)	Bullying victimization was reported more frequently in high school cases than in middle or elementary school cases; reports varied by informant type.	
Looijmans et al. (2021) [55]	The Netherlands	Data-set 1	Multi-methods psychological autopsy study	Qualitative informant interviews	Parents and peers	School and cyberbullying addressed in informant interviews.	Yes		Suicide deaths	10–19	16	2017			Discrepancies between informants were reported regarding the presence of bullying victimization.	
Mérelle et al. (2019) [54]	The Netherlands	Data-set 1	Multi-methods psychological autopsy study	Qualitative informant interviews; questionnaires.	Parents, siblings, peers, teachers, employers; healthcare professionals	School and cyberbullying assessed via questionnaires and informant interviews.	Yes		Suicide deaths	10–19	35	2017		47% overall; 22% cyber-bullying	Interview reports described emotional distress and perceived peer rejection; bullying was reported in the months preceding death for some LGB youth.	
Neider et al. (2016) [81]	Sweden		Mixed-methods cohort study	Clinical assessments and informant interviews	Parents and close relatives	Childhood bullying assessed in interviews	Yes		Suicide deaths of patients with schizo-phrenia	18–48	12	1979–2012		55%	Bullying victimization was reported more frequently in the suicide group. No association was reported between bullying victimization and dopamine or serotonin levels in cerebrospinal fluid.	
Van Bergen et al. (2025) [57]	The Netherlands	Data-set 1	Multi-methods psychological autopsy study	Qualitative informant interviews	Parents, peers, siblings, teachers	School and cyberbullying addressed in informant interviews.	Yes		Suicide deaths of adolescent girls	10–19	18	2017			Among girls who died by suicide, bullying victimization and revictimization were reported within identified life trajectories, including stigmatization related to sexual orientation.	
**LONGITUDINAL SURVEY STUDIES**																
Geoffrey et al. (2014) [64]	United Kingdom	Data-set 4	Prospective population-based birth cohort study	Birth survey and follow-up assessment at age 7; questionnaires; standardized tests of reading and mathematics	Mothers and teachers	Peer bullying reported by mothers at age 7	Yes		Suicide deaths	Base-line: 7	Total cohort: 16,470 Suicide deaths: 51	1958–2009		12–31% (frequency-dependent) among suicide deaths; 4–24% in total sample	Bullying victimization assessed at or before age 7 was associated with suicide mortality (sometimes: HR 1.36; frequently: HR 2.84).	
Geoffrey et al. (2023) [63]	United Kingdom	Data-set 4	Prospective population-based birth cohort study	Follow-up assessments at ages 7 and 11; questionnaires; standardized tests of reading and mathematics	Mothers and teachers	Peer bullying reported by mothers at ages 7 and 11	Yes		Suicide deaths	Base-line: 7–11	Total cohort: 14,946 Suicide deaths: 55	1958–2012		23–34% (frequency-dependent) among suicide deaths; 18–28% in controls	Bullying victimization was associated with suicide mortality (OR 1.29), with attenuated estimates after adjustment for sex and individual and familial vulnerabilities (OR 1.18).	
**ONLINE ETNOGRAPHY STUDIES**																
Fratini & Hemer (2020) [85]	Online		Exploratory qualitative case study	Qualitative analysis of live-broadcasted suicide cases	Individuals broadcasting suicidal behavior and interacting online audiences	Audience responses coded as bullying victimization.	No		Suicide deaths and suicide attempt	18–20	2/1	2016–2019			Audience responses during live-broadcasted suicides were reported as cyberbullying.	
Ma et al. (2016) [84]	China		Online ethnographic multiple case study	Qualitative analysis of online reports and digital records	Online audiences and media sources documenting live-broadcasted suicides	Audience responses coded as bullying victimization.	No		Suicide deaths and suicide attempts	19–24	1 (subset of 6 cases)	2014			Audience responses during live-broadcasted suicides were reported as cyberbullying; direct interaction with viewers was observed in some cases.	

With the exception of the population-based longitudinal birth cohort studies, bullying victimization was assessed retrospectively based on informant report or self-report. For longitudinal cohort studies, bullying prevalence is reported for suicide cases and the reference population; for all other studies, frequencies refer to suicide cases only

The included studies varied in research scope, methodology, design, and, consequently, the level of detail provided regarding bullying victimization. Studies were categorized according to design and methodological approach, resulting in the following classification (see Table 2):

A. *Record-based studies*: studies using death certificates, coroner’s reports, and/or police records, sometimes supplemented by interviews with family, suicide notes or other written materials produced by the young person who died by suicide (19 studies);
B. *Predominantly qualitative narrative studies*: studies based predominantly on qualitative interviews or open-ended survey responses from bereaved individuals (9 studies);
C. *Longitudinal survey studies*: studies in which individuals who later died by suicide had completed surveys at several time points from childhood through adolescence (2 studies);
D. *Online ethnography studies* (2 studies).

Several studies were based on the same dataset (Dataset 1: Mérelle et al. [54], Looijmans et al. [55], Balt et al. [56], Van Bergen et al.; [57] Dataset 2: Rodway et al.; [58–60] Dataset 3: Freuchen et al.; [61, 62] and Dataset 4: Geoffroy et al. [63, 64]). Not all studies reported both the frequency and the role of bullying victimization in relation to suicide. Some addressed only the frequency (*N* = 7), others examined only the role of bullying victimization (*N* = 7), whereas 18 studies examined both aspects.

### Assessment of bullying victimization

The first category of studies (A, record-based studies) is characterized by large sample sizes and reliance on official records. The number of individuals who died by suicide ranged from 36 to 9,884 (total *N* = 21,469), with a mean of 1,130 and a median of 429.

Within this category, bullying victimization was not systematically assessed but was identified only when explicitly documented in official reports or mentioned by informants. As a result, the identification of bullying victimization depended heavily on the sources of information available.

The types and combinations of data sources varied across record-based studies. Some relied on a single type of official record, whereas others combined multiple sources [58–60, 65–71], including coroner’s reports [72], police records [73], and information obtained from healthcare professionals or health services [74] In addition, several studies supplemented official records with information from bereaved individuals [75–77], witnesses [78], and/or materials originating from the deceased, such as suicide notes [79, 80].

Across 16 record-based studies [58–60, 65–77], bullying victimization was not the primary focus and can therefore be considered an incidental finding. These studies primarily examined suicides within specific populations and explored life adversities and broader suicide risk factors. Three record-based studies adopted a more targeted focus on bullying victimization: one examined a range of interpersonal conflicts that included bullying [78], another assessed whether experiences of bullying victimization constituted a contributing factor in youth suicide cases [79], and a third investigated the prevalence of bullying victimization among LGBTQ youth [80].

Uncertainty regarding the assessment of bullying victimization was common within this category. Nine studies did not specify how bullying victimization was assessed [58–60, 65, 66, 72, 73, 76, 80], and the respective authors were therefore contacted for clarification. Four of them responded, indicating that bullying victimization had been identified only when explicitly mentioned by an informant or professional in the available data sources. This limitation was also acknowledged in the literature itself, with one study explicitly noting that all stressors and clinical variables, including bullying victimization, should be regarded as estimates subject to potential underreporting [79].

The second category of studies (B, predominantly qualitative narrative studies) is characterized by smaller sample sizes than record-based studies (A), greater heterogeneity in study populations, and substantial variability in the number and type of informants. The number of suicide cases in predominantly qualitative studies (B) ranged from 12 to 417 (total *N* = 633), with a mean of 70 and a median of 35.

Within category B, data collection relied on multiple informants, with considerable variation both between and within studies. In Dataset 1 [54–57], various combinations of informants were used – such as parents, peers or siblings, employers or teachers, and healthcare professionals – and the number of informants per case varied [54–57].

In contrast to record-based studies, bullying victimization was generally assessed systematically in predominantly qualitative studies. It was typically addressed as a standard topic in qualitative interviews and/or questionnaires covering sociodemographic characteristics and life experiences, including exposure to bullying victimization during childhood [81]. However, exceptions to systematic assessment were observed within this category. In some studies, bullying victimization was recorded only when explicitly mentioned by a teacher completing a student suicide case report [82], or it remained unclear whether open-ended questions explicitly addressed bullying victimization [83]. For example, one study [83] identified a theme related to “experiences of bullying, racism, and discrimination,” but it was unclear whether bullying victimization had been explicitly assessed in the questionnaire.

The third category of studies (C, longitudinal survey studies) comprised large-scale prospective cohort designs with repeated assessments across childhood. These studies enabled examination of early-life exposures – such as low birth weight, young maternal age, externalizing behavioral problems, cumulative emotional adversity, and bullying victimization – in relation to later suicide outcomes. Across this category, the study population was drawn from a nationally representative birth cohort followed from early childhood into adulthood. Sample sizes were substantial: analyses included 16,470 participants at age 7 [64] in one assessment and 14,946 participants at ages 7 and 11 in another [63], reflecting attrition over time. Despite the large sample sizes, the number of suicide deaths observed during follow-up remained low. At the time of the earlier analysis, 51 participants had died by suicide [64], increasing to 52 participants in the later analysis [63].

Within category C, bullying victimization was assessed prospectively in a systematic and standardized manner through maternal reports. Mothers completed standardized survey questionnaires when their children were aged 7 years [63, 64] and, in one study, again at age 11 years [63]. They reported how frequently their child had been bullied by other children using predefined response categories (“never,” “sometimes,” or “frequently”).

The fourth category of studies (D, online ethnography studies) comprised two qualitative investigations analyzing online content relating to individual suicide cases. Across both studies, four suicide deaths and five suicide attempts were described, of which three suicide deaths met the age-based inclusion criteria.

Within category D, bullying victimization was not directly measured. Instead, the original authors interpreted specific audience interactions as instances of cyberbullying. Behaviors such as inciting or derogatory comments (e.g., “You deserve to die” or “Why are you still alive?”), public endorsement through “liking” suicidal posts, and other hostile responses were classified as indicative of cyberbullying within their analytical frameworks [84, 85]. As no predefined or standardized definition of bullying was applied and the raw data were not accessible, identification of cyberbullying relied entirely on the original authors’ interpretations.

### Frequency of bullying victimization in adolescent suicide cases

#### A. Record-based studies

In 5.6% of all suicide cases included in the record-based studies, bullying victimization was mentioned as a factor or adversity present in the lives of individuals who died by suicide.

Gender emerged as a predictive factor in the association between bullying and suicide. Five record-based studies distinguished between male and female suicide cases [58–60, 67, 77]. In these studies, bullying victimization was mentioned in 4.3% of male cases compared with 12.2% of female cases. Age also had predictive value in relation to bullying and suicide findings. Four record-based studies categorized suicide cases by age group [60, 69, 77, 80]. In three of these studies [69, 77, 80], participants were divided into three overlapping age categories: approximately 5–13 years (youngest group), 10–18 years (middle group), and 15–19 years (oldest group). Overall, bullying victimization was more frequently reported among younger adolescents who died by suicide (up to approximately 16 years of age), with a prevalence of 17.5%, compared with 2.6% among older adolescents.

Sexual and gender diversity also emerged as an important factor in studies examining the association between bullying victimization and suicide outcomes. Three record-based studies distinguished between LGBTQ+ youth and non-LGBTQ+ youth [66, 71, 80]. Bullying victimization was more frequently reported among LGBTQ+ youth who died by suicide (17.4%) than among their non-LGBTQ+ peers (4.7%).

Regarding the role of online abuse vis-à-vis traditional forms of bullying, four studies [58–60, 74] – three of which were part of the same UK-wide investigation of adolescent suicides [58–60] – distinguished between cyberbullying and face-to-face bullying. Face-to-face bullying was more prevalent than cyberbullying, with reported rates of 12.9% and 4.4%, respectively. Notably, in most cases where cyberbullying occurred, face-to-face bullying was also present (55.2%) [59].

#### B. Studies based predominantly on qualitative research

Across predominantly qualitative narrative studies, bullying victimization was identified as a contributing factor in a variable proportion of suicide cases, ranging from 3.1% to 55% [54, 56, 61, 62, 81, 82]. In retrospective interview studies with bereaved individuals specifically, reported proportions ranged from 29% to 55% [54, 56, 61, 62, 81].

Within this body of research, distinctions were made between different forms and contexts of bullying victimization. Several publications were based on the same underlying dataset; however, only two of these explicitly reported frequency-related information on bullying victimization. In the original mixed-methods study, Mérelle et al [54] reported that 47% of participants mentioned bullying, and 22% indicated being bullied via the internet. Balt et al [56] subsequently analyzed qualitative interview data from the same cohort and described four instances of cyberbullying. They distinguished cyberbullying – defined as the repeated communication of harmful or hostile messages intended to cause harm – from broader cybervictimization, which encompassed online harassment and other intrusive behaviors. Consistent with the definition adopted in the present review, only cyberbullying was included. As both publications drew on the same sample, these findings are reported descriptively and were not aggregated in order to avoid double counting.

We observed variability in the reporting of bullying victimization, influenced by contextual and demographic factors. In one study [61], the availability of suicide notes was associated with higher reporting rates. In 38.9% of cases in which adolescents left a suicide note, bullying victimization was mentioned either in the note itself and/or by bereaved individuals during interviews. By contrast, when no suicide note was available, bullying victimization was reported in 21% of cases by bereaved individuals. In one study [82], gender differences in prevalence were observed, with bullying victimization reported in 2.9% of male adolescents and 3.5% of female adolescents who died by suicide.

#### C. Longitudinal survey studies

Within longitudinal survey studies, bullying victimization was reported relatively frequently by informants. Across both studies [63, 64] – drawing on data from a nationally representative birth cohort – bullying victimization was mentioned by mothers in 43.1–45.5% of cases. Specifically, bullying victimization was reported as occurring sometimes in 15–31% of cases and frequently in 10–12% of cases [63, 64], reflecting graded assessments of exposure collected prospectively through standardized survey items.

#### D. Online ethnography studies

In the online ethnography studies (D), it was not possible to calculate the frequency of bullying victimization.

### The role that bullying victimization can play in relation to suicide

#### A. Record-based studies

In most record-based studies, bullying victimization was either not identified in the cases examined or was reported too infrequently to permit firm conclusions about its contribution. For example, one study reported that bullying victimization was identified as a motive for suicide only in extremely rare cases [73], while others reported bullying victimization in only a small proportion of cases [65, 78], precluding conclusions about its specific role in the suicide process.

In those studies in which inferences about the role of bullying victimization were possible, it appeared to function within a constellation of multiple risk factors rather than as an isolated cause. Several studies suggested that bullying co-occurred with other psychosocial stressors and adversities. Furthermore, bullying victimization was more frequently reported among LGBTQ+ youth, female adolescents, and younger individuals who died by suicide than among their non-LGBTQ+, male, or older counterparts [60, 66, 67, 77, 80]. These findings suggest that bullying victimization may act as a particularly salient stressor or contributing factor within specific subgroups of youth.

Evidence from detailed case-based analyses further illustrates the heterogeneity in the role of bullying victimization. In one study, youth deaths initially classified as suicides were subsequently reclassified as definite suicides, undetermined deaths, and non-suicides, with case vignettes used to contextualize these distinctions. In one vignette describing a case classified as a definite suicide, bullying victimization appeared to function as a trigger [75]. However, the extent to which this observation can be generalized to the broader sample remains unclear. By contrast, findings from another study indicated that bullying victimization reflected enduring psychosocial stress rather than serving as an immediate trigger for suicide [60]. Youth responses to bullying victimization varied substantially across individuals and contexts, with some adolescents demonstrating relative resilience and others exhibiting heightened vulnerability to its psychological sequelae [72]. In a small number of extreme cases, witnesses identified bullying victimization as the principal precipitating factor in adolescent suicide [72].

#### B. Studies based predominantly on qualitative research

Across predominantly qualitative narrative studies, bullying victimization emerged as a salient factor in the psychological well-being of young people; however, its precise role in suicide deaths remains difficult to delineate. Informants suggested that bullying victimization may contribute to enduring psychological distress and exacerbate established suicide-related risk factors, including feelings of sadness, lack of belongingness, and entrapment [54, 56, 57]. In one study focusing on adolescent female suicide deaths, bullying victimization was associated with a progressive deterioration in mental health during adolescence, manifesting in low self-esteem, social mistrust, and self-harming behaviors [57].

At the same time, studies in this category indicated that bullying victimization was not uniformly identified as a direct or singular antecedent of suicide. Rather, its influence appeared embedded within a broader constellation of psychosocial vulnerabilities. Nevertheless, a subset of bereaved parents or peers explicitly attributed the suicide death to prior traumatic experiences of bullying victimization [56]. Particularly among LGBQ girls, bullying related to sexual orientation appeared to function as a more proximal stressor in the period preceding suicide. These findings suggest that bullying victimization may operate both as a long-term vulnerability factor and, under certain circumstances, as an acute precipitating influence [57].

The available literature in category B further suggests that the role of bullying victimization in relation to suicide is conditional on the presence and course of psychopathology [62, 81]. Childhood bullying victimization appeared to play a role in the pathways leading to later suicide among individuals with specific mental health trajectories, including those who developed schizophrenia in adulthood [81] or subthreshold depressive symptoms [62]. These findings point to an interaction between experiences of bullying victimization and individual mental health trajectories in shaping suicide risk, rather than exerting a simple additive effect of bullying victimization.

One study emphasized that bullying victimization in the context of subsequent suicide is embedded within broader patterns of institutional unsafety and social marginalization [83]. Retrospective parental accounts described their children’s pervasive experiences of bullying as intertwined with discrimination related to race, sexual orientation, gender identity, and mental health status, alongside perceived inadequacies in school and healthcare responses.

Finally, several studies highlighted methodological challenges in retrospectively reconstructing bullying victimization prior to suicide. Some divergences between parental and peer reports regarding the presence and severity of bullying underscore the difficulty of establishing a coherent account of pre-suicide experiences and complicate efforts to examine the role of bullying in adverse outcomes following death [55].

#### C. Longitudinal survey studies

Longitudinal population-based research drawing on nationally representative birth cohorts indicates that frequent bullying victimization in childhood is associated with an increased risk of suicide death in adulthood. One study demonstrated that frequent bullying victimization was prospectively associated with suicide death over a follow-up period of up to five decades, with stronger associations observed for frequent victimization (*HR* = 1.36, 95% CI 0.72–2.56 for sometimes bullied and *HR* = 2.84, 95% CI 1.16–6.96 for frequently bullied) [64]. A second study based on the same cohort demonstrated that this association was attenuated after adjustment for a wide range of individual and familial vulnerabilities, including childhood emotional and behavioral problems, cognitive ability, socioeconomic disadvantage, maternal age at birth, and adverse childhood experiences within the family (unadjusted *OR* = 1.29, 95% CI 1.02–1.64; fully adjusted *OR* = 1.18, 95% CI 0.92–1.51) [63].

Taken together, findings from category C suggest that bullying victimization operates only marginally, if at all, as an independent predictor of suicide death. Rather it appears to contribute modestly to suicide risk within a broader constellation of interrelated developmental and psychosocial vulnerabilities.

#### D. Online ethnography studies

In online ethnography studies, audience reactions extended beyond supportive or rescue-oriented engagement and frequently took the form of cynical, indifferent, or overtly hostile behaviors. These responses included mockery; questioning the authenticity of suicidal intent (“doing it for fame”); symbolic endorsement through “liking” suicidal posts; explicit encouragement of the act (“you deserve to die”); and the provision of denigrating or procedural “advice” on how to carry out the act “properly.” [85]. Within these online environments, specific audience roles were identified that can be conceptualized as bullying actors. Interventions by so-called “Suicide Experts” involved cynical or humiliating guidance on how to commit suicide, while “Trolls” engaged in overt hostility, baiting, and explicit encouragement of self-harm [84]. These behaviors were consistently described as exploiting the acute vulnerability of suicidal individuals and reinforcing experiences of hopelessness, dehumanization, and humiliation [84, 85].

Studies suggest that these online dynamics may heighten the perceived obligation by suicidal youth to complete their act, intensify suicidal determination, or – if the attempt is unsuccessful – leave lasting psychological harm. Moreover, hostile reactions from online audiences can transform an acute crisis into a public spectacle, shaping both the decision to follow through with the act and the meanings subsequently ascribed to it by observers [84, 85].

## Discussion

This scoping review examined the role of bullying victimization in relation to suicide deaths among children and adolescents. Two research questions guided the review. The first asked how frequently bullying victimization in childhood and adolescence is reported in youth suicide cases. The findings indicated that bullying victimization was not systematically investigated in most of the included studies [58–60, 65–76, 78–80, 82, 84, 85]. In record-based studies, it was documented only when mentioned by relatives, peers, or professionals during investigations of the death by coroners, police or healthcare professionals. When bullying victimization was systematically examined – primarily in psychosocial autopsy studies and longitudinal survey studies – reported prevalence varied substantially by study design, ranging from 29% to 55% in retrospective qualitative autopsy studies [54, 62, 81] and from 43.1% to 45.5% in longitudinal survey studies [63, 64]. These methodological differences may therefore have contributed to an underestimation of prevalence rates of bullying victimization among young people who died by suicide.

The second objective of this review was to examine the role of bullying victimization as a risk factor or potential pathway to suicide among young people. Overall, the findings align with previous studies indicating that bullying victimization contributes to suicide risk in interaction with other clinical, psychosocial, and environmental risk factors [4, 14, 15]. In the reviewed literature, bullying victimization is most often mentioned, either as one of multiple co-occurring influences (primarily in quantitative studies [58–60, 65–78) or as a contextual factor (primarily in qualitative studies [54–57, 61, 62, 64, 81–85). Evidence indicates that bullying victimization often occurs alongside other psychosocial adversities, including school problems [76], sexual abuse [56, 57, 74], family problems [76], mental health diagnoses [57, 62, 81], and difficulties in access to appropriate mental health care [57, 83].

Age, gender, and sexual orientation appear highly relevant contextual factors, as younger adolescents [66, 70, 77], girls [58, 60, 67, 77], and LGBTQ+ youth [54, 57, 58, 66, 71, 80] who died by suicide are particularly likely to have experienced bullying victimization. Qualitative and case-based studies describe how bullying victimization elicited feelings of social exclusion, rejection, and persistent not belonging [54, 56, 57], particularly among LGBTQ+ youth [57, 60, 66, 77, 80]. Several studies further report that bullying related to sexual orientation or gender identity was embedded in broader experiences of marginalization, isolation, and stigmatization in school and peer contexts [83], often accompanied by feelings of loneliness, social mistrust, and diminished self-worth [54–57].

Across study designs, these co-occurring social, psychiatric, and psychological adversities were frequently associated with low self-esteem, self-injurious behaviors, and broader psychosocial distress [54–57], suggesting that bullying victimization may contribute indirectly to suicide risk through an accumulation and interaction of vulnerabilities. Importantly, longitudinal studies demonstrated that frequent bullying victimization in childhood was associated with a small but elevated risk of suicide death later in life, even after controlling for individual and familial vulnerabilities [63, 64]. This prospective evidence from cohort studies indicates that bullying victimization can act as a longer-term vulnerability embedded within developmental trajectories, rather than solely as an immediate trigger preceding suicide [86]. Although reported less frequently, retrospective findings from psychological autopsy studies and suicide note analyses suggest that, in some cases, bullying victimization may act as a proximal trigger or tipping point, particularly among LGBTQ + and other minoritized youth [57, 61].

These observed patterns align with key constructs from leading theoretical frameworks of suicide. For example, the IPS [18] posits thwarted belongingness and perceived burdensomeness as central drivers of suicidal desire. Although the studies reviewed did not explicitly operationalize these constructs, narratives describing bullying-related exclusion, rejection, and chronic social alienation suggest that bullying victimization may intensify feelings of thwarted belongingness along pathways to suicide. Similarly, the IMVM [19] and the Cry of Pain model [20] highlight defeat and entrapment as precursors within the motivational phase of suicidal ideation. These concepts resonate with findings from the reviewed studies. Informants suggested that bullying victimization may contribute to persistent psychological distress and exacerbate established suicide-related risk factors, including feelings of sadness, lack of belongingness, and entrapment [54, 56, 57]. In this sense, repeated experiences of bullying may foster perceptions of social defeat and diminished self-worth, which, when combined with limited coping resources or perceived avenues of escape, can develop into feelings of entrapment.

### Strengths and limitations

This scoping review has several strengths. First, it uniquely focuses on suicide deaths among children and adolescents – an outcome rarely examined in the bullying literature, which has predominantly addressed suicidal ideation and nonfatal attempts. Second, by integrating evidence across diverse study designs – including record-based studies, qualitative narratives, longitudinal cohort studies, and online ethnographies – the review provides a comprehensive and nuanced synthesis of how bullying victimization has been examined in relation to suicide death. Third, the review explicitly describes how bullying victimization was assessed across studies, demonstrating that its prevalence in suicide cases is likely underestimated when it is not actively and systematically investigated.

In addition to these contributions, several methodological limitations of this scoping review should be acknowledged. First, substantial heterogeneity in study designs, combined with a relatively small number of included studies, limits the generalizability of our findings. Second, although the qualitative studies provided rich and detailed insights and were more likely to include systematic accounts of bullying victimization, their methodological quality was constrained by small sample sizes and limited generalizability, Third, since only a few studies employed a longitudinal design, it remains difficult to assess causality and temporality in relation to bullying victimization.

Several study-specific limitations further illustrate the methodological challenges in the field. For example, difficulties in case classification were evident across studies. The lack of a clear distinction between suicide and accidental death – including drug-related fatalities – raises concerns about outcome misclassification [72]. Similarly, in livestream-based ethnographic analyses, the absence of a systematic distinction between fatal suicides and nonfatal attempts limits confidence in case ascertainment and comparability across cases [84, 85]. The assessment of bullying was not always optimal, as information was often obtained retrospectively from family members [54–57, 62, 75–81, 83] or peers [54–57, 75–77, 80], who may not have been fully objective informants. For example, discrepancies emerged in how negative peer interactions were perceived: bereaved relatives tended to interpret them as bullying, whereas teachers and classmates described them as ordinary disputes [72]. In addition, some studies did not clearly specify whether bullying referred exclusively to victimization or also included perpetration [78]. Finally, minor inconsistencies in analytic reporting limited interpretability in some cases, such as the presentation of word frequencies without corresponding case numbers [82] and an unexplained discrepancy in the reported number of suicide cases included in the bullying analysis [81].

### Implications

The findings of this scoping review highlight the need to strengthen both the empirical and theoretical foundations of research on bullying victimization and youth suicide. Future studies should adopt more rigorous and diverse methodological approaches, including longitudinal population-based studies and the systematic and standardized assessment of bullying victimization. Moreover, given the expansion of cyberbullying and its presumed harmful effects [10, 27], there is a pressing need to develop methodologies that facilitate a comprehensive understanding of the digital environment.

At a theoretical level, the findings suggest that broad models of suicide – such as the IPS - , may offer a useful conceptual framework for understanding cases in which bullying victimization plays a prominent role. However, these models have rarely been applied specifically to bullying-related suicide. Future research should therefore prioritize in-depth, fine-grained case-based and mixed-method approaches to examine how adequately existing suicide theories explain the specific mechanisms and developmental pathways observed in these cases.

Beyond research implications, the findings are also relevant for policy and practice, particularly in the areas of suicide prevention and digital platform governance. By demonstrating that bullying victimization often functions as a longer-term vulnerability embedded within broader psychosocial adversity, this review highlights the need for suicide prevention strategies that emphasize early identification and continuity of care. At the same time, the growing relevance of cyberbullying underscores the importance of platform-level policies that address harmful online behavior, livestreamed content, and audience interactions that may exacerbate suicidal risk.

## Conclusion

This scoping review examined the role of bullying victimization in relation to suicide deaths among children and adolescents. Across a heterogeneous and methodologically constrained body of literature, bullying is reported in 29–55% of youth suicide cases when assessed systematically. However, it was rarely the sole focus of inquiry and was often likely underreported.

The findings support three key conclusions. First, bullying victimization appears to be embedded within broader constellations of adversity. Second, it can be identified retrospectively as an early-life or background stressor. Third, in a limited number of cases it may function as a proximal precipitating factor – most commonly among young people who are stigmatized because of their identities or in cases where the suicide was publicly broadcast on social media platforms.

Progress in this field will require longitudinal, and methodologically rigorous research that explicitly and systematically integrates bullying victimization into theoretical models of the pathways to suicide. In particular, greater attention should be directed towards cyberbullying and the influence of online interactions, including social media harassment and livestreamed content. A more comprehensive understanding of how current social environments shape pathways from vulnerability to fatal suicidal behavior is essential for strengthening the scientific basis of youth suicide prevention.

## Supplementary Information

Supplementary Material 1

## Data Availability

All data generated or analyzed during this study are included in this published article and its supplementary materials. Additional information, including data extraction materials, is available from the corresponding author upon reasonable request.

## Abbreviations

AI: Artificial Intelligence
CASP: Critical Appraisal Skills Programme
CI: Confidence Interval
CINAHL: Cumulative Index to Nursing and Allied Health Literature
ERIC: Education Resource Information Center
HR: Hazard Ratio
IMVM: Integrated Motivational–Volitional Model
IPS: Interpersonal Theory of Suicide
LGBQ: Lesbian, Gay, Bisexual and Queer
LGBTQ+: Lesbian, Gay, Bisexual, Transgender, Queer and other sexual and gender diverse identities
MEDLINE: Medical Literature Analysis and Retrieval System Online
NCIS: National Coronial Information System
NFR-CRS: National Fatality Review-Case Reporting System
NVDRS: National Violent Death Reporting System
ONS: Office for National Statistics
OR: Odds Ratio
QUIPS: Quality In Prognosis Studies
SDQ: Strengths and Difficulties Questionnaire
SocINDEX: Sociology Index
UK: United Kingdom
WHO: World Health Organization
